# Treatment Resistant Depression Rates and Related Clinical Variables in a Outpatient Clinic: Pilot data from Türkiye

**DOI:** 10.1192/j.eurpsy.2025.673

**Published:** 2025-08-26

**Authors:** S. Karabulut, M. Düzgün

**Affiliations:** 1 Psychiatry, Akdeniz University School of Medicine, Antalya, Türkiye

## Abstract

**Introduction:**

Major depressive disorder is a global mental health challenge imposing a serious burden on individuals and society. Treatment resistant depression (TRD) is most commonly defined as inadequate treatment response following at least two consecutive antidepressant trials of adequate dose, duration and treatment adherence. TRD is associated with high personal suffering, considerable functional impairment and significant mental health costs.

**Objectives:**

This study investigated the rate of TRD in treatment-seeking outpatients with major depressive disorder and clinically-related variables. We hypothesized that patients with TRD would have more severe symptoms, chronic course and more hospitalization than patients without TRD.

**Methods:**

The files of patients diagnosed with Major Depressive Disorder who had applied to the Outpatient Psychiatry within the last 3 months and had a follow-up history of at least 1 year were reviewed (n=204). Demographic and clinical data of the patients were recorded using a structured data form. Dutch Measure for quantification of Treatment Resistance in depression (DM-TRD) scores were calculated. The study was approved by the Akdeniz University Ethics Committee (approval date:22/8/2024).

**Results:**

Regarding the index episode, the majority of patients had received selective-serotonin reuptake inhibitor (SSRI) treatment (22% escitalopram, 14.2% sertraline). 30.9% of the patients had received augmentation. After the first treatment trial, 21.1% of the patients had treatment-response, and 30.7% achieved remission. After the second treatment trial, 10.5% showed treatment-response, and 16.3% achieved remission. The proportion of patients meeting the criteria for TRD was 29.9%.

When comparing patients with TRD, the total number of depressive episodes was significantly lower (p=0.01), the duration from the onset of the index episode to treatment and recovery was longer (p<0.001, p=0.02; respectively) in those with TRD. The number of ECT episodes and rTMS sessions was higher (p<0.001, p=0.004; respectively), the DM-TRD score, the frequency of benzodiazepine use and the rate of inpatient treatment were also higher (p<0.001, p<0.001, p=0.01; respectively). The rate of non-adherence to treatment, the rate of chronic episodes and symptom severity were higher, functional impairment was more severe, and the frequency of comorbid personality disorders was higher (p<0.001, all).

**Conclusions:**

To the best of our knowledge, this is the first study reporting TRD data from Türkiye. Our results showed that patients with TRD had more chronic and less repetitive illness course, duration of untreated depressive symptoms were longer, use of benzodiazepine, ECT and rTMS treatments were more frequent and longer than patients without TRD. These results should alert clinicians about subprofiles of patients with TRD to predict course and develop preventive effective strategies.

**Disclosure of Interest:**

None Declared

